# HlSRB, a Class B Scavenger Receptor, Is Key to the Granulocyte-Mediated Microbial Phagocytosis in Ticks

**DOI:** 10.1371/journal.pone.0033504

**Published:** 2012-03-29

**Authors:** Kyaw Min Aung, Damdinsuren Boldbaatar, Rika Umemiya-Shirafuji, Min Liao, Naotoshi Tsuji, Xuan Xuenan, Hiroshi Suzuki, Aiko Kume, Remil Linggatong Galay, Tetsuya Tanaka, Kozo Fujisaki

**Affiliations:** 1 Department of Pathological and Preventive Veterinary Science, The United Graduate School of Veterinary Science, Yamaguchi University, Yoshida, Yamaguchi, Japan; 2 Laboratory of Emerging Infectious Diseases, Department of Frontier Veterinary Medicine, Faculty of Agriculture, Kagoshima University, Korimoto, Kagoshima, Japan; 3 Laboratory of Parasitic Diseases, National Institute of Animal Health, Kanondai, Tsukuba, Ibaraki, Japan; 4 National Research Center for Protozoan Diseases, Obihiro University of Agriculture and Veterinary Medicine, Inada, Obihiro, Japan; University of Minnesota, United States of America

## Abstract

Ixodid ticks transmit various pathogens of deadly diseases to humans and animals. However, the specific molecule that functions in the recognition and control of pathogens inside ticks is not yet to be identified. Class B scavenger receptor CD36 (SRB) participates in internalization of apoptotic cells, certain bacterial and fungal pathogens, and modified low-density lipoproteins. Recently, we have reported on recombinant HlSRB, a 50-kDa protein with one hydrophobic SRB domain from the hard tick, *Haemaphysalis longicornis*. Here, we show that HlSRB plays vital roles in granulocyte-mediated phagocytosis to invading *Escherichia coli* and contributes to the first-line host defense against various pathogens. Data clearly revealed that granulocytes that up-regulated the expression of cell surface HlSRB are almost exclusively involved in hemocyte-mediated phagocytosis for *E. coli* in ticks, and post-transcriptional silencing of the HlSRB-specific gene ablated the granulocytes' ability to phagocytose *E. coli* and resulted in the mortality of ticks due to high bacteremia. This is the first report demonstrating that a scavenger receptor molecule contributes to hemocyte-mediated phagocytosis against exogenous pathogens, isolated and characterized from hematophagous arthropods.

## Introduction

The ixodid ticks (Arthropoda: Ixodidae), popularly known as hard ticks, serve as a unique vector of various pathogens that cause deadly diseases, such as Lyme disease, tick-borne encephalitis, Rocky Mountain spotted fever, babesiosis, theileriosis, and anaplasmosis, during hematophagy. Ticks are only second to mosquitoes as vectors of various pathogens that cause deadly diseases of human and animals [1]. Scavenger receptors are cell-surface proteins and exhibit distinctive ligand-binding properties, recognizing a wide range of ligands that include microbial surface constituents and intact microbes. It is of current interest to look at the molecular scenario, in particular, the role of scavenger receptor in hemocyte-mediated phagocytosis, inside vector ticks, which play an important role in first-line host defense against invading pathogens.

Phagocytosis refers to the recognition, engulfment, and intracellular destruction of invading pathogens and apoptotic cells by individual hemocytes [2]. Phagocytosis in mammals is mainly achieved by mononuclear phagocytic cells, such as macrophages and dendritic cells and polynuclear neutrophils [3]. In arthropods, such as insects [4] and ticks [5], [6], phagocytosis is achieved mainly by the circulating plasmatocytes and/or granulocytes, in the hemolymph. Since phagocytosis is a widely conserved cellular process that occurs in many protozoa and all metazoans, it could be hypothesized that arthropod phagocytosis is also similar to mammalian phagocytosis [4], [7], [8]. However, the molecular mechanisms of hemocyte-mediated phagocytosis in arthropods have not been intensively investigated [4].

The class B scavenger receptor CD36 (SRB), the cell surface glycoprotein, is present on a variety of cell types, including insect hemocytes [8], [9]. SRB has been implicated in a wide variety of cellular processes, including fatty acid transport and regulation of angiogenesis [10], [11]. Recent findings provide evidence for the essential role of SRB as a pattern-recognition receptor mediating innate immune responses of the mammalian [3] and insect hosts [4] to a range of exogenous pathogens. Other findings by Baranova et al. [12] indicated that human SRB serves as a phagocytic receptor for a variety of bacteria and mediates pathogen-induced JNK-mediated signaling (c-Jun NH_2_-Terminal kinase-mediated signaling). However, the role of arthropod SRB documented in the hemocyte-mediated phagocytosis to invading pathogens has been very restricted to *Drosophila* [4], [8], and the precise functions of SRB in the uptake of various microbes into hemocytes are largely unknown, particularly in hematophagous and vector arthropods, such as ticks and mosquitoes.

In our previous study, the gene encoding putative class B scavenger receptor (designated as *HlSRB*) was identified and characterized from the ixodid tick, *Haemaphysalis longicornis* [13]. The HlSRB had overall 30% identity to both mammalian and insect SRB membrane proteins. The mRNA transcripts of *HlSRB* were expressed in multiple organs of adult females but with varying levels in the different developmental stages of ticks. The recombinant HlSRB was expressed in *Escherichia coli* as His-tagged protein, and anti-mouse recombinant HlSRB serum elucidated the localization of endogenous protein in the midgut, salivary gland, and ovary of partially fed *H. longicornis* female ticks. Gene silencing of *HlSRB* in female ticks illustrated the significant reduction of engorged body weights and egg production [13]. In addition, we demonstrated for the first time that the class B scavenger receptor CD36 may not only mediate the uptake of exogenous dsRNAs in ticks but also play essential roles for systemic RNAi of ticks [14].

Here, we demonstrate that *HlSRB-*specific gene-silenced ixodid ticks, such as *H. longicornis*, completely lost the phagocytic ability of their hemocytes, in particular, granulocytes, to combat an exogenous bacterial pathogen, Gram-negative *E. coli*, and consequently failed to efficiently clear bacterial burdens in hemolymph and survive due to the profound bacteremia. To the best of our knowledge, HlSRB is the first scavenger receptor molecule contributed to hemocyte-mediated phagocytosis against exogenous bacteria, isolated and characterized from hematophagous arthropods.

## Materials and Methods

### Ticks and animals

The parthenogenetic Okayama strain of the ixodid tick *H. longicornis* has been maintained by feeding on Japanese white rabbits, *Oryctolagus cuniculus* (Kyudo, Kumamoto, Japan) in our laboratory [15]. Rabbit care was approved by the Animal Care and Use Committee of Kagoshima University (Approval no. A08010).

### Preparation of unfed (UF), partially fed (PF), and *HlSRB* dsRNA-injected female (RNAi-tick) ticks and microinjection of *E. coli* into these tick groups

Three groups of ticks, i.e., unfed female (UF) 270 ticks, partially fed female (PF) 270 ticks, and *HlSRB* dsRNA-injected female (RNAi-tick) 270 ticks, were used in this experiment. In our study, “UF”, “PF”, and “RNAi-tick” indicate the abbreviations of the “unfed female ticks”, “partially fed female ticks” and “*HlSRB* dsRNA-injected female ticks”.

UF ticks were maintained at 15°C in an incubator. To obtain PF ticks, UF ticks were fed on Japanese white rabbits, and, 3 days after attachment, ticks were collected as PF ticks. For RNAi-ticks, the *HlSRB* dsRNA was injected into UF ticks (total 0.5 µl; 1 µg/tick). The dsRNA injection was followed as described previously [13]. *HlSRB* dsRNA was injected into the ticks, through the fourth coxae into the haemocoel; the ticks were fixed on a glass slide with adhesive tape. The injections were carried out using 50-µl microcapillaries (MICROCAP®, Drummond Scientific, Broomall, PA, USA) drawn to fine-point needles. The needles were connected to an air compressor. Injected ticks were infested on the ears of rabbits 24 hours after injection [13]. Four days after infestation, ticks were removed and collected from rabbits, and three ticks were subjected to RT-PCR to determine whether or not the *HlSRB* gene was silenced [13].

The UF, PF, and RNAi-ticks were left at 25°C in an incubator for subsequent experiments. The injection of heat-killed *E. coli* (72°C for 1.5 mins [16]), *E. coli*, or *HlSRB* dsRNA into ticks and construction of *HlSRB* dsRNA were performed as described previously [13].

An *E. coli* (pathogenic strain O157) was grown in a Luria-Bertani broth medium (BD, Sparks, MD, USA) at 37°C. When the optical density at 600 nm reached 0.5 (OD_600_ = 0.5), *E. coli* cells were induced with 1 mM isopropyl *β*-D-1-thiogalactopyranoside (IPTG) and incubated for another 4 hours. An *E. coli* suspension was respectively injected (0.5 µl/tick) to UF, PF, and RNAi-ticks groups through the fourth coxae into the haemocoel. Control ticks were injected with an equal volume of heat-killed *E. coli* (0.5 µl/tick). The similar numbers of heat-killed *E. coli* or *E. coli* were injected into three groups of ticks. The injected ticks were left to rest at 25°C in an incubator for 24 hours, and hemolymph collection was then performed as described below.

### Preparation of hemolymph and hemocyte samples

Hemolymph samples from UF, PF, and RNAi-ticks injected with or without *E. coli* or heat-killed *E. coli* were collected by amputating the forelegs of female ticks at the coxal-trochanteral joint, drawn into heparinized capillary tubes containing 100 µl of PBS [5], [17], [18], and then loaded to Shandon EZ Double Cytofunnel (Thermo Electron Corp., Milford, MA, USA). Hemocyte smears were obtained from these hemolymph samples by using a Cytospin 4 cytocentrifuge machine (Thermo Electron) at 100 *g* for 5 minutes, and smears on the micro-glass slides were then air-dried and fixed in methanol or cold acetone for 10 minutes. Smears fixed in methanol were immediately stained with a 3% Giemsa solution for 30 minutes for the light microscopic examination of hemocyte morphology and counting of granulocyte population, and other smears fixed in cold acetone or unfixed micro-glass slides were kept at −80°C until use for IFAT.

### Indirect immunofluorescent antibody test (IFAT)

To examine endogenous HlSRB localization in hemocytes from UF, PF, and RNAi-ticks, hemocyte smears on the micro-glass slides described above were blocked with 5% skim milk in PBS overnight at 4°C and incubated for 30 minutes at 37°C with 1∶100 dilution of an anti-rHlSRB (rHlSRB) mouse serum [13] as a primary antibody. After washing three times with PBS, Alexa 488-conjugated goat anti-mouse immunoglobulin (1∶1000; Invitrogen) was applied as secondary antibody at 37°C for 1 hour. After washing three times with PBS, hemocyte smears were mounted in a mounting medium with DAPI (Vectashield, Vector Laboratories, Burlingame, CA, USA) and then covered with a cover glass. The images were photographed and recorded using a fluorescence microscope (Olympus, Tokyo, Japan).

### Tick survival monitoring

The changes with time in the survival rates of UF, PF, and RNAi-ticks after injection with heat-killed *E. coli* or *E. coli* were monitored using different tick groups from the ticks for hemocyte examinations. Preparation of *E. coli* incubation and injection were followed as described above. Female ticks injected with heat-killed *E. coli* or *E. coli* were left at 25°C in an incubator and their survival was checked every 6 hours for 2 days after injection. In this experiment, a total of 150 ticks were injected for heat-killed *E. coli* or *E. coli*: 25 ticks for heat-killed *E. coli* and 25 ticks for *E. coli* injection in each group. This survival monitoring was performed at least in triplicate.

### Examination of population of phagocytic hemocytes after *E. coli* injection

To examine the populational changes of phagocytic hemocytes after *E. coli* injection, hemolymph samples (5 µl/tick) of Pf ticks 3, 12, 24, and 48 hours after injection with heat-killed *E. coli* or an *E. coli* suspension were prepared for hemocyte smears, as described above. Plasmatocytes and granulocytes were counted in a 3% Giemsa solution (5 ticks/group), as described above, using a light microscope (Olympus).

### Reverse transcriptase-polymerase chain reaction (RT-PCR)

To investigate the expression pattern of the *HlSRB* gene after *E. coli* injection, hemolymph samples of PF ticks 3, 12, 24, and 48 hours after injection with heat-killed *E. coli* or an *E. coli* suspension were subjected to total RNA extraction using the TRIzol reagent (Invitrogen, CA, USA). The RT-PCR analysis was performed using a one-step RNA PCR kit (Takara, Otsu, Japan) with the primer sets of the *HlSRB* [13] gene. Control amplification was carried out using the *H. longicornis β-actin*-specific primers (accession no. AY254898). The PCR products were subjected to electrophoresis in a 1.5% agarose gel in a TAE buffer; the DNA was visualized by ethidium bromide staining and analyzed using Quantity One 1-D Analysis Software (Quantity One Version 4.5, Bio-Rad Laboratories, Milan, Italy).

### Protein expression analysis by Western blotting

Hemolymph samples of PF ticks 3, 12, 24, and 48 hours after injection with heat-killed *E. coli* or *E. coli* suspension were separated by SDS-polyacrylamide gel electrophoresis and transferred to a polyvinylidene difluoride membrane (Millipore, Bedford, MA, USA). The membrane was blocked with 5% skim milk in PBS-T (137 mM NaCl, 2.7 mM KCl, 10 mM Na_2_HPO_4_, 1.8 mM KH_2_PO_4_, 0.05% Tween-20, pH 7.4) and then incubated with 1∶100 dilution of anti-rHlSRB or 1∶200 dilution of anti-actin serum [19] as a primary antibody. After the incubation of peroxide-conjugated sheep anti-mouse IgG as a secondary antibody (1∶2000 dilution; GE Healthcare, Little Chalfont, UK), the specific protein bands were detected using 0.5 mg/ml 3, 3′-diaminobenzidine tetrahydrochloride.

### Construction of recombinant *E. coli* expressing green fluorescent protein (EGFP)

A 759-bp DNA fragment containing an open reading frame of the enhanced green fluorescent protein (EGFP) gene was isolated from pEGFP (Clontech, Palo Alto, CA, USA) and inserted into the *Sal* I-*Not* I sites of the *E. coli* expression vector, pRSET-B (Invitrogen) as recommended by the manufacturer. The resulting plasmid was designated as pRSET-B/EGFP.

An *E. coli* DH5α strain-competent cells (Invitrogen), the same biological effects and phenotype as for *E. coli* O157 [20], colony transformed with pRSET-B/EGFP was cultured in an LB broth medium (BD) supplemented with 50 µg/ml of ampicillin. When the optical density at 600 nm reached 0.5, *E. coli* cells were induced to express the recombinant EGFP by the addition of 1 mM IPTG and incubation for another 4 hours.

### Injection and culture of EGFP-expressing *E. coli*

EGFP-expressing *E. coli* (E-*E. coli*), readily detectable by microscopy [21], was injected as a suspension (0.5 µl) into UF, PF, and RNAi-ticks, respectively. The injected ticks were left for 24 hours at 25°C in an incubator, and hemolymph samples were then collected by amputating the forelegs of female ticks at the coxal-trochanteral joints. One drop of hemolymph was placed on a glass slide and covered with a cover glass, and the hemocyte images were photographed using a fluorescence microscope (Olympus).

Five microliters of hemolymph from UF, PF, or RNAi-ticks 24 hours after E-*E. coli* injection was applied to an LB agar medium including 1 µg/ml of ampicillin and 100 mM/plate of IPTG. All emerged colonies were counted within 24 hours using FluorChem FC2 (Cell Biosciences, California, USA). In addition, the emerged colonies were observed under UV light in order to confirm whether they were E-*E. coli* or not [21].

### Statistical analyses

All statistical analyses were done with the Student's *t*-test. *P*<0.05 values were considered significant.

## Results

### Plasmatocytes and granulocytes of female ticks

**Figure 1:**
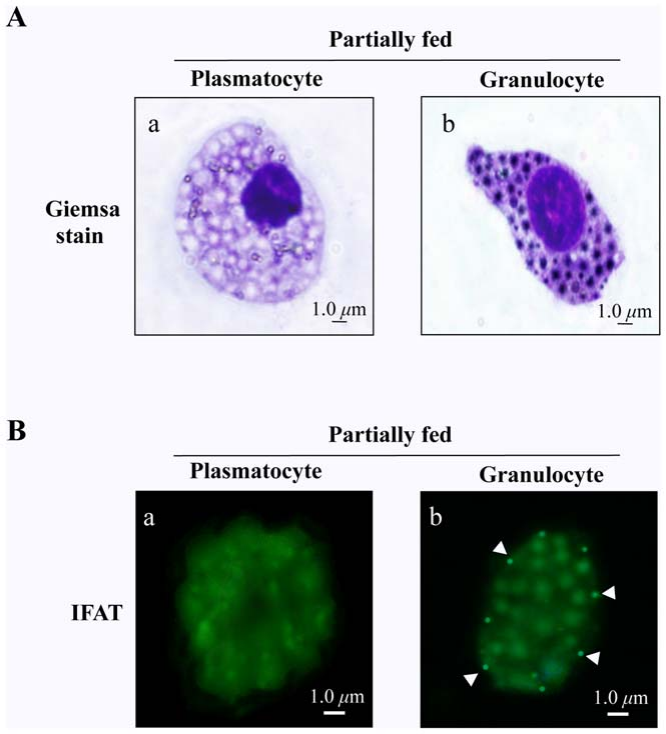
Giemsa-stained and localization of HlSRB on the plasmatocytes and granulocytes without heat-killed *E. coli* or *E. coli* injection. 3% Giemsa-stained plasmatocytes and granulocytes (A) and localization of the endogenous HlSRB on the surface of granulocyte from partially fed female *H. longicornis* adult ticks (PF) by IFAT (B). Hemolymph and hemocyte samples were prepared as indicated in Materials and methods. The hemocytes were stained with anti-rHlSRB antibody followed by Alexa 488-conjugated mouse anti-IgG. Phagocytic plasmatocytes and granulocytes were observed under fluorescence microscopy. Arrowheads indicate the native HlSRB expressed on the surface of granulocytes. Typical plastmatocytes (a) and granulocytes (b) are shown. The *scale bar* represents 1 µm.

The current observations were performed on plasmatocytes and granulocytes of female *H. longicornis* ticks (Fig. 1A), since they are generally recognized as a predominant class of phagocytes circulating in hemolymph of ticks [6], [21]–[23]. Plasmatocytes of ticks have a round or irregular shape with processes such as filopodia, and they have few granules in the cytoplasm. The granulocytes of ticks show a spherical form and are filled with many large granules [6], [21], [24].

An immunohistochemical examination using an indirect fluorescent antibody test (IFAT) was conducted to illustrate the localization of the endogenous HlSRB protein in the phagocytic plasmatocytes and granulocytes of PF ticks without heat-killed *E. coli* or *E. coli* injection (Fig. 1B). IFAT was performed using anti-rHlSRB mouse serum followed by Alexa 488-conjugated anti-mouse immunoglobulin. As shown in Fig. 1B, the localization of native HlSRB protein was detected only on the surface of granulocytes, while no localization was observed in plasmatocytes (Fig. 1B), suggesting that endogenous HlSRB is expressed predominantly in granulocytes of *H. longicornis*.

### Morphological changes of plasmatocytes and granulocytes of female ticks after *E. coli* injection

**Figure 2:**
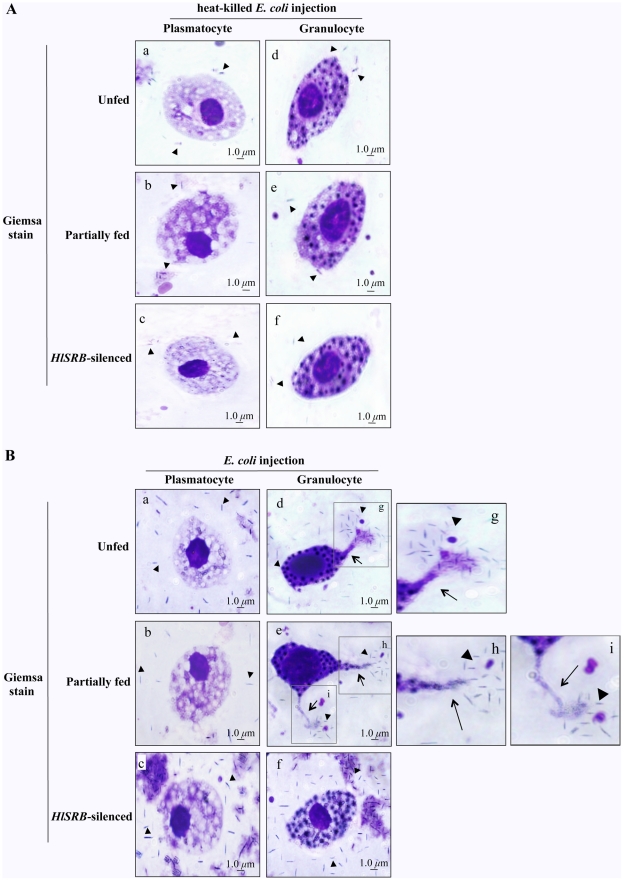
Giemsa-stained plasmatocytes and granulocytes after heat-killed *E. coli* or *E. coli* injection. 3% Giemsa-stained plasmatocytes and granulocytes from *H. longicornis* unfed (UF), partially fed (PF), and *HlSRB* dsRNA-injected ticks (RNAi-ticks) 24 hours after the injection with heat-killed *E. coli* (A) or *E. coli* (B). Heat-killed *E. coli* or *E. coli* was percutaneously injected into UF, PF, and RNAi-ticks. The injected ticks were left at 25°C in an incubator. Twenty-four hours after injections, hemolymph was collected for the examination of hemocytes. Arrowheads indicate *E. coli*, and arrows indicate the lobopodia-like extensions of granulocytes. Plasmatocytes and granulocytes of UF (a and d), PF (b and e), and RNAi-ticks (c and f). Areas marked by squares are shown at higher magnification (g, h, and i). The *scale bar* represents 1 µm.

We examined the morphological changes of two types of phagocytic hemocytes, plasmatocytes and granulocytes, in UF, PF, and RNAi-tick groups 24 hours after heat-killed *E. coli* or *E. coli* injection (Fig. 2). Based on our observation of Giemsa-stained smears, no changes were observed in the shape of plasmatocytes and granulocytes from UF, PF, and RNAi-ticks 24 hours after heat-killed *E. coli* injection (Fig. 2A). Similarly, there were no remarkable morphological changes in plasmatocytes from UF, PF, and RNAi-ticks 24 hours after *E. coli* injection (Fig. 2B, panel a, b, and c). Interestingly, large lobopodia-like structures [24] were observed in granulocytes of the Giemsa-stained smears from UF and PF ticks 24 hours after *E. coli* injection (Fig. 2B, panel d, and e, arrows), and many *E. coli* bacteria were found around the top of lobopodia-like structures (Fig. 2B, panel g, h, and i). However, no lobopodia-like structures were detected in granulocytes from RNAi-ticks after *E. coli* injection (Fig. 2B, panel f). According to our results of Fig. 2A and B, the different amount of extracellular heat-killed *E. coli* or *E. coli* were found in the hemolymph because heat-killed *E. coli* could not grow and *E. coli* could grow after 24 hours injection at 25°C.

### Localization of endogenous HlSRB proteins expressed in granulocytes of female ticks after *E. coli* injection

**Figure 3:**
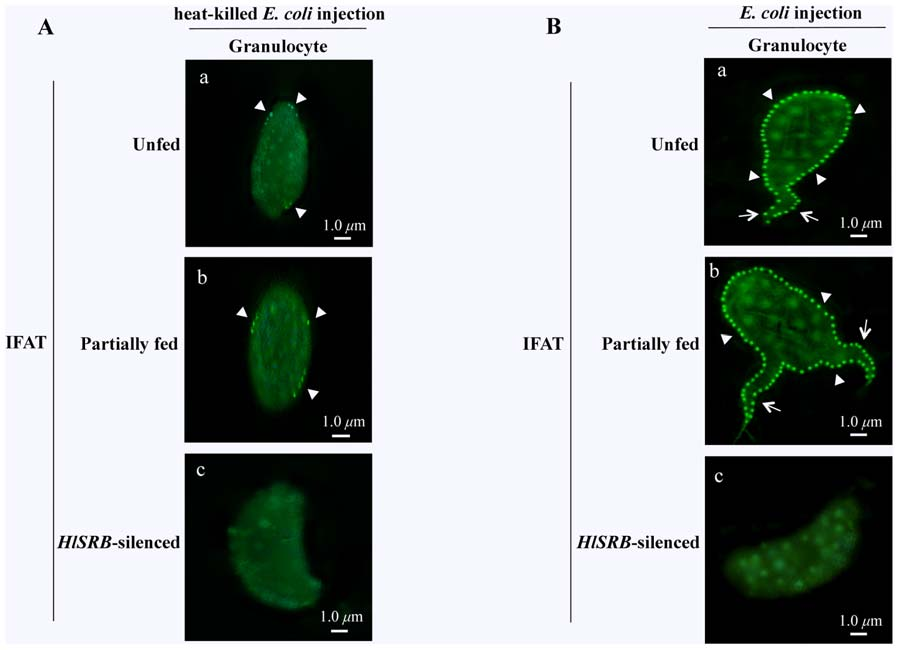
Immunohistochemical localization of the endogenous HlSRB on the surface of granulocytes from heat-killed *E. coli*- (A) or *E. coli*-injected (B) unfed (UF), partially fed (PF), and *HlSRB* dsRNA-injected ticks (RNAi-ticks) by IFAT. The IFAT experiment was performed as shown in Fig. 1. Arrowheads indicate the native HlSRB expressed on the surface of granulocytes, and arrows indicate the lobopodia-like extensions of granulocytes. Granulocytes of UF (a), PF (b), and RNAi-ticks (c). The *scale bar* represents 1 µm.

The precisely ultrastructural localization of human SRB has shown on the surface of the hepatic sinusoidal lining cells, hepatic microvilli, and microvilli of (HepG2-A16) hepatoma cells and C32 amelanotic melanoma cells [25]. Barnwell et al. [26] reported that human SRB was localized on the surface of C32 melanoma cells, giving a pattern of bright and spotty fluorescence. In our study, we observed the immunohistological localization of endogenous HlSRB proteins in granulocytes 24 hours after heat-killed *E. coli* or *E. coli* injection. As shown in Fig. 3A, there was a few HlSRB localization, around 8 in number, on the surface of granulocytes from UF and PF ticks 24 hours after heat-killed *E. coli* injection (panel a, and b), while no positive fluorescence was observed on the surface of granulocytes from RNAi-ticks injected with *E. coli* (panel c) by IFAT. In addition, no morphological changes were observed in the granulocytes from UF, PF, and RNAi-ticks injected with heat-killed *E. coli* (Fig. 3A).

A significantly large number of fluorescent dots showing localization of native HlSRB protein were observed on almost all the surface of granulocytes from UF and PF ticks 24 hours after *E. coli* injection (Fig. 3B, panel a, and b), and they were 60 to 90 in number and uniformly distributed throughout the surface of granulocytes including lobopodia-like structures. However, no positive fluorescence was detected on the surface of granulocytes from RNAi-ticks injected with *E. coli* (Fig. 3B, panel c). These data suggest that granulocytes of *H. longicornis* ticks might morphologically respond to *E. coli* injection and up-regulate the expression of cell surface HlSRB, but HlSRB-silenced granulocytes might have failed to respond properly to *E. coli* invasion in hemolymph.

### Survival rates of female ticks after *E. coli* injection

**Figure 4:**
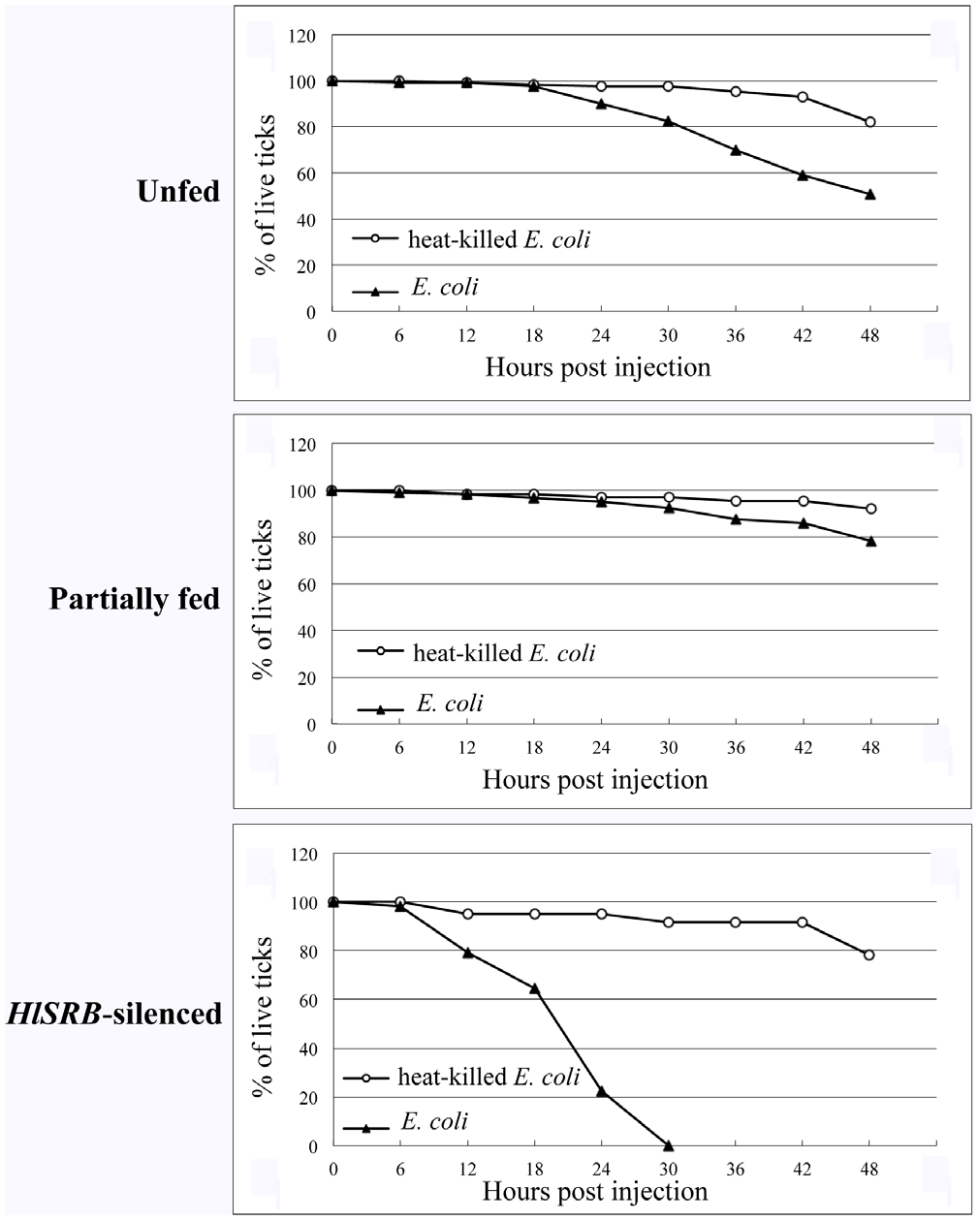
Survival rate comparisons among unfed (UF), partially fed (PF), and *HlSRB* dsRNA-injected ticks (RNAi-ticks) after heat-killed *E. coli* or *E. coli* injection. Heat-killed *E. coli* or *E. coli* was percutaneously injected into UF, PF, and RNAi-ticks. The injected ticks were allowed to rest at 25°C in an incubator and then monitored for survival rate. The survival rates were calculated by the percentage of remaining live ticks to the number of ticks used at the beginning of the experiment in different time courses. The moribund ticks were calculated as dead ticks. The figures are shown to represent data in combined results of three different experiments.

High survival rates were consistently observed in UF, PF, and RNAi-ticks after heat-killed *E. coli* injection (Fig. 4). The survival rates of UF, PF, and RNAi-ticks 48 hours after heat-killed *E. coli* injection were 82.1%, 92.1%, and 78.2%, respectively (Fig. 4), indicating that changes with time in survival rates were only modest in ticks injected with heat-killed *E. coli* and no significant differences were obvious among UF, PF, and RNAi-ticks. However, the survival rates of female ticks injected with *E. coli* were quite different between UF and PF ticks and RNAi-ticks. The survival rates of UF and PF ticks 30 and 48 hours after *E. coli* injection were 82.5 to 92.5% and 50.8 to 78.3%, respectively (Fig. 4), showing only a slight decrease with time. However, the survival rates of RNAi-ticks 18, 24, and 30 hours after *E. coli* injection were 64.1%, 22.5%, and 0%, respectively (Fig. 4), indicating a marked decrease with time. These results indicated that almost all UF and PF ticks could survive after proper control of invaded *E. coli* but RNAi-ticks had to succumb to *E. coli* burdens mainly due to the recognition failure in pathogen-associated molecular patterns (PAMPs) [4], [27] caused by infection due to high bacteremia.

### Populational changes of plasmatocytes and granulocytes of female ticks after *E. coli* injection

**Figure 5:**
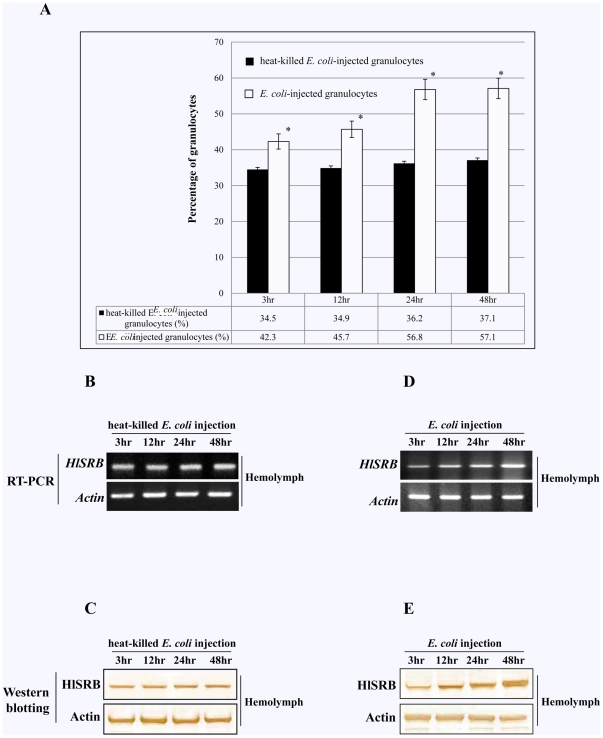
Populational changes of granulocytes and expression patterns of *HlSRB* gene and endogenous HlSRB protein in the hemolymph from partially fed (PF) ticks injected with either heat-killed *E. coli* or *E. coli*. PF ticks were percutaneously injected with heat-killed *E. coli* or *E. coli*. The injected ticks were left at 25°C in an incubator. Hemolymph was collected for granulocyte counts, RNA extraction, and preparation of protein lysates in different time courses after heat-killed *E. coli* or *E. coli* injection. Effect of *E. coli* injection on granulocyte population (A). Black and white bars indicate heat-killed *E. coli* and *E. coli* injection, respectively. Values represent the mean ± SD of five ticks. The asterisks indicate a significant difference from the control heat-killed *E. coli* injections (*P*<0.05). RT-PCR analysis (B and D). PCR was performed using cDNA synthesized from the Pf ticks injected with either heat-killed *E. coli* or *E. coli* with primer sets specific to *HlSRB* and *β-actin* gene. Western blot analysis (C and E). Hemolymph samples were subjected to SDS-PAGE under reducing conditions and transferred to a PVDF membrane. The membrane was probed with the mouse anti-rHlSRB or mouse anti-actin serum was used as a control. 3 hr, 3 hours after heat-killed *E. coli* or *E. coli* injection; 12 hr, 12 hours after heat-killed *E. coli* or *E. coli* injection; 24 hr, 24 hours after heat-killed *E. coli* or *E. coli* injection; 48 hr, 48 hours after heat-killed *E. coli* or *E. coli* injection.

We examined changes in population of phagocytic hemocytes from PF ticks after *E. coli* injection in different time courses. Based on our observation of Giemsa-stained smears, the population of plasmatocytes and granulocytes increased after *E. coli* injection. In this study, we focused on the population of granulocytes because HlSRB was expressed only in the granulocytes (Figs. 1B and 3). As shown in Fig. 5A, the population of granulocytes were increased with a different time course not only slightly in ticks after heat-killed *E. coli* injection but also more significantly in ticks injected with *E. coli*. These percentages represent, on average, the results of five ticks from each group. This result suggests that the increased granulocytes population might be the result of granulocyte-mediated phagocytosis for invasion foreign microorganisms and that the slightly increase is related to possible external injuries caused by microinjections in recipient ticks.

### Expression patterns of HlSRB in hemolymph of PF ticks after *E. coli* injection

The expression patterns of HlSRB in hemolymph from PF ticks after *E. coli* injection were examined by RT-PCR and Western blot analysis in different time courses. As shown in Fig. 5B and C, the β-actin gene and protein levels did not change in ticks injected with heat-killed *E. coli* or *E. coli*. However, the gene and protein expressions of HlSRB were up-regulated with time slightly in ticks after heat-killed *E. coli* injection and more significantly in ticks injected with *E. coli* (Fig. 5D and E), suggesting that HlSRB expression in tick hemolymph containing various types of hemocytes and humoral proteins [6], [28] might be up-regulated for participation in both immunological defense against *E. coli* and wound-healing after microinjection.

### Key roles of HlSRB in granulocyte-mediated phagocytosis to *E. coli*

**Figure 6:**
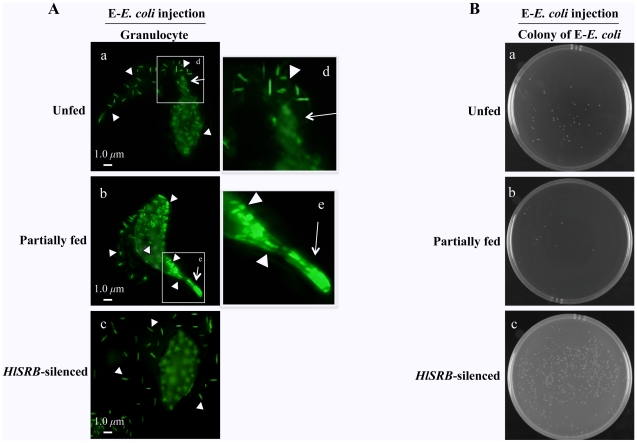
Fluorescence microscopy showing the fluorescence of EGFP-expressing *E. coli* (E-*E. coli*) in granulocytes (A) and colony number of E-*E. coli* propagated in unfed (UF), partially fed (PF), or *HlSRB* dsRNA-injected ticks (RNAi-ticks) (B). UF, PF, and RNAi-ticks were injected with E-*E. coli*. Twenty-four hours after the injection, hemolymph was collected from these ticks by amputation of legs. One drop of hemolymph placed on a glass slide was examined under fluorescence microscopy (A). Arrowheads indicate E-*E. coli* and arrows indicate the lobopodia-like extensions of granulocytes of UF (a), PF (b), and RNAi-ticks (c). Areas marked by squares are shown at higher magnification (d and e). The *scale bar* represents 1 µm. Colony numbers of E-*E. coli* propagated in UF, PF, and RNAi-ticks 24 hours after E-*E. coli* injection (B). Hemolymph of these tick groups was applied on an LB agar medium, and the number of emerged colonies of *E. coli* after overnight culture was counted. This experiment was done in triplicate, and similar results were obtained in 3 different experiments.

Hemolymph samples were collected from UF, PF, and RNAi-ticks 24 hours after injection with EGFP-expressing *E. coli* (E-*E. coli*) and examined under fluorescence microscopy (Fig. 6A). In UF and PF ticks injected with E-*E. coli*, granulocytes were found to contain many phagocytosed bacteria and appeared to extend a lobopodia-like structure toward a colony of bacteria (panel a, b, d, and e). In RNAi-ticks injected with E-*E. coli*, bacteria accumulated around granulocytes, and no phagocytosed bacteria were observed in the cytoplasm of granulocytes (panel c). These results suggested that HlSRB expression of granulocytes up-regulated in response to *E. coli* invasion (Fig. 3B) might reflect a critical role of HlSRB in hemocyte-mediated phagocytosis to invading *E. coli* and also that lobopodia-like extension of granulocytes might be specialized for bacteria clearance in hemolymph.

### Culture of hemolymph from ticks injected with EGFP-expressing *E. coli* (E-*E. coli*)

The colony numbers of E-*E. coli* after overnight cultivation of hemolymph from UF, PF, and RNAi-ticks 24 hours after E-*E. coli* injection were 88, 21, and 971, respectively (Fig. 6B). These numbers are shown to represent in average results of three different experiments and a significantly highest number of colonies was observed in culture of hemolymph from RNAi-ticks. These results suggested that RNAi-ticks induced high bacteremia in hemolymph 24 hours after E-*E. coli* injection due to the failure in combating bacteria, caused by knockdown of HlSRB, resulting in the emergence of a large number of colonized bacteria.

## Discussion

Previous literature on hemocyte identification of arthropods suggests that the most common types of hemocytes are prohemocytes, plasmatocytes, granulocytes, and spherulocytes [2], [6], [8], [17], [18], [29]. At least two types of hemocytes, plasmatocytes and granulocytes, are generally recognized as a predominant class of phagocytic hemocytes circulating in hemolymph of insects [2], [8], [30] and ticks [5], [22]–[24], [31], [32]. Other finding by Ceraul et al. [33] showed that encapsulation/nodulation may be an important component of the immune response in ticks after direct inoculation of *E. coli* bacteria into the hemocoel cavity. Cattle are the main reservoir for *E. coli* O157 inducing hemorrhagic enteritis [34] and *H. longicornis* is one of the important parasite for the cattle. In the present study, we provide evidence that granulocytes of *H. longicornis* ticks after *E. coli* injection show overt populational and morphological changes, such as an increased number of granulocytes and an extension of lobopodia-like structures toward a colony of *E. coli*. We also demonstrated that phagocytosed EGFP-expressing *E. coli* (E-*E. coli*) was found only inside the granulocytes. These results strongly suggest that granulocytes are almost exclusively involved in hemocyte-mediated phagocytosis for *E. coli* in *H. longicornis* ticks.

Ticks must acquire nutrients from the host blood meal and metabolize these nutrients via metabolism [35]. After blood feeding, increase in phagocytosis takes place in the hemolymph of fed ticks compared with their unfed ticks state [6]. In *Ornithodoros moubata* soft tick, the population of hemocytes corresponding increase in fed ticks showed increase eosinophilic granulocytes populations and increase phagocytic activity in fed ticks than their unfed ticks [18]. According to our results of UF's hemolymph (Fig. 2B, panels a and d; Fig. 6A, panel a), the amount of extracellular *E. coli* or E-*E. coli* were higher than those of PF's hemolymph (Fig. 2B, panels b and e; Fig. 6A, panel b), while the highest in RNAi-tick's hemolymph (Fig. 2B, panels c and f; Fig. 6A, panel c), suggest that phagocytic activity of PF ticks is higher than those of UF ticks and loss of phagocytic activity in RNAi-ticks.

It was shown in this study that the gene and protein expressions of HlSRB [13] are significantly up-regulated in tick hemolymph after *E. coli* injection. In addition, the fluorescent dots showing localization of native HlSRB, detected only on the surface of granulocytes, demonstrated a marked 10-fold increase after *E. coli* injection. These results indicate that granulocytes up-regulate the expression of cell surface HlSRB in response to exposure to *E. coli*, most likely resulting in increased HlSRB in hemolymph.

Our *in vivo* gene silencing study revealed that HlSRB-specific gene-silenced ticks were unable to properly control invaded *E. coli* burdens and had to succumb to high bacteremia. Interestingly, in HlSRB-silenced ticks, no fluorescent dots showing HlSRB localization were detected in granulocytes before and after *E. coli* injection, and lobopodia-like structures and intracellularly phagocytosed E-*E. coli* bacteria were not observed. It was indicated that the mammalian SRB generally implicates as a sensor of microbial products that mediate phagocytosis in response to a broad range of pathogens [12]. Therefore, our findings prompted us to speculate that HlSRB is critically involved in the uptake of *E. coli* bacteria into granulocytes and thus HlSRB silencing resulted in the complete loss of the granulocyte-mediated phagocytosis, giving rise to the mortality of ticks after *E. coli* injection.

The current study raises the possibility that the phagocytosis of tick granulocytes is induced when HlSRB is activated by target pathogens. However, phagocytosis of a microbe by a phagocytic cell is an extremely complex and diverse process which requires multiple successive interactions between the phagocyte and the pathogen as well as sequential signal transduction events [2], [4], [8]. Cytokine-related molecules such as PDGF-AB, TNF-α and IL-8 in invertebrates are known to provoke conformational changes in mollusk hemocytes and to affect phagocytosis [7], [36]. Scavenger receptors expressed by mammalian myeloid cells have been elucidated to alter cell morphology, and their expression is affected by various cytokines [37]. We have already shown in *Ornithodoros moubata* ticks that granulocytes have platelet-derived growth factor (PDGF)-AB [32]. In invertebrate immunocyte, PDGF-AB caused changes in cellular shape via interactions with the respective receptors [38]. Therefore, it may be assumed that the participation of PDGF-AB in the elongation of lobopodia-like structures in tick granulocytes after *E. coli* injection is caused in cooperation with HlSRB. Further studies should be carried out in order to clarify the molecular cooperation between HlSRB and PDGF-AB in granulocyte-mediated phagocytosis in ticks.

Collectively, HlSRB, a class B scavenger receptor CD36 of ixodid ticks, is found to play a key role in granulocyte-mediated phagocytosis to invading *E. coli* and contribute to the first-line host defense against various pathogens. These findings indicate that HlSRB may be critical for the survival of ixodid ticks. Furthermore, our data suggest that HlSRB may be a novel promising target molecule for the development of vaccine against ticks and tick-borne diseases.
